# Supplementary material for: A deep neural network model of audiovisual speech recognition reports the McGurk effect
Source: Psychon Bull Rev. 2026 Feb 18;33(2):84. doi: 10.3758/s13423-025-02846-8 (PMC12917083; doi:10.3758/s13423-025-02846-8)
Supplement: Supplementary file 2 — (HTML 1.15 MB) [file 13423_2025_2846_MOESM2_ESM.html]

A Deep Neural Network Model of Audiovisual Speech Recognition Reports the McGurk Effect


Code 

- Show All Code
- Hide All Code

# A Deep Neural Network Model of Audiovisual Speech Recognition Reports the McGurk Effect

#### H. Ma, et al

- 1 Running the code in this
  document
  - 1.1 Code setup
  - 1.2 Functions used
- 2 Load up AVHuBERT and Human
  data
- 3 McGurk Stimuli
  - 3.1 Plot veridical vs. non-veridical
    responses
- 4 Congruent stimuli
  - 4.1 Auditory only ba
- 5 Change from Ao to AV
- 6 Comparison with Humans
  - 6.1 McGurk stimuli
    - 6.1.1 Non-veridical responses
    - 6.1.2 Number of unique
      phonemes
    - 6.1.3 Compare frequency of
      non-veridical response types
  - 6.2 Congruent performance
  - 6.3 Incongruent non-McGurk

# 1 Running the code in this document

Scroll down to see the results of the analysis. To run the analysis
code yourself, please install R (R Studio is also recommended) and copy
and paste the code blocks into the R (Studio) console. The code blocks
(in grey) are cumulative and must be run in sequence. Your code output
should match the output in the HTML file.

The first block of code below attempts to install and then load
several R libraries. All of the libraries are on CRAN and should be
available for any recent version of R. If you are prompted to install
source versions that require compilation, you can respond “no” and just
get the most recent binary versions (compiling from source requires
access to other tools).

## 1.1 Code setup

```
suppressMessages(sapply(c('lme4', 'car', 'readxl', 'magrittr', 'stringr', 'knitr', 'emmeans', 'dplyr', 'tidyr'), function(nm) {
    if (!require(nm, quietly = TRUE, character.only=TRUE)) {
        install.packages(nm)
        
        Sys.sleep(0.125)
        if(!require(nm, quietly = TRUE, character.only = TRUE)) {
            stop(paste("Could not load package: ", nm, ". Try again later, make sure your internet connection is stable."))
        }
    }
}))
```

## 1.2 Functions used

```
se <- function(x, na.rm=TRUE) {
    n <- sum(!is.na(x))
    
    if(n==0) return(NA)
    if(n==1) return(0)
    
    stats::sd(x, na.rm=na.rm) / sqrt(n)
}

m_se <- function(x, na.rm=TRUE) {
    c(
        'mean' = mean(x, na.rm=na.rm),
        'se' = se(x, na.rm=na.rm)
    )
}

fix_pdf_name <- function (fname)  {
    if (!grepl("\\.pdf$", fname)) {
        fname = paste0(fname, ".pdf")
    }
    return(fname)
}

# TEST = TRUE means write to current plotting device rather than opening a PDF
as_pdf <- function (fname, w, h, expr, TEST = TRUE, bg = "white") 
{
    if (!TEST) {
        on.exit(dev.off())
        fname <- fix_pdf_name(fname)
        pdf(fname, width = w, height = h, useDingbats = FALSE, 
            bg = bg)
        res = eval(expr)
    }
    else {
        res = eval(expr)
    }
    return(invisible(res))
}


# kable doesn't like matrix output from aggregate
fix_agg_columns <- function(m) {
    to_fix <- which(sapply(m, is.matrix))
    for(ii in seq_along(to_fix)) {
        mat = m[[to_fix[ii]]]
        for(v in colnames(mat)) {
            m[[paste0(names(to_fix)[ii], '_', v)]] = mat[,v]
        }
    }
    m[to_fix] = NULL
    return(m)
}


do_aggregate <- function(x, ...) {
    return(stats::aggregate(data = x, ...))
}

rbind_list <- function(ll) do.call(rbind, ll)

ebars <- function (x, y, sem, length = 0.05, up = T, down = T, code = 0, ...) {
    
    if (up) {
        arrows(x0 = x, y0 = as.numeric(y), y1 = as.numeric(y + 
                                                               sem), angle = 90, code = code, length = length, ...)
    }
    if (down) {
        arrows(x0 = x, y0 = as.numeric(y), y1 = as.numeric(y - 
                                                               sem), angle = 90, code = code, length = length, ...)
    }
}


which.equal <- function(x,y) {
    which(x==y)
}

ebar_polygon <- function (x, y, sem, alpha = 100, col = "black", fill = col, 
                          stroke = col, border = NA, add_line = TRUE, lwd = 1, ...) 
{
    is_finite = is.finite(y) & is.finite(sem)
    if (all(!is.finite(sem))) {
        is_finite <- is.finite(y)
        sem <- 0 * y
    }
    x = x[is_finite]
    y = y[is_finite]
    sem = sem[is_finite]
    sem = abs(sem)
    polygon(c(x, rev(x)), c(y + sem, rev(y - sem)), border = border, 
            col = getAlphaRGB(fill, alpha))
    if (add_line) 
        lines(x, y, col = stroke, lwd = lwd, ...)
}

plus_minus <- function (x, d) {
    if (missing(d) & is.matrix(x)) {
        d <- x[, 2]
        x <- x[, 1]
    }
    c(x - d, x + d)
}

density_jitter <- function(x, around=0, max.r=.2, n=length(x), seed=NULL) {
    if(n < 2) {
        return(around)
    }
    dx <- density(x, from=min(x), to=max(x), n=n)
    
    # careful here, switching from x to y (i.e., y = f(x))
    y <- approxfun(dx)(x)
    
    pts <- R.utils::withSeed({
        runif(length(x),
              min = -max.r * (y/max(y)),
              max = max.r * (y/max(y))
        ) + around
        
    }, seed = seed)
    
    return(list(
        points=pts,
        density=dx,
        y=y
    ))
}


dark_colors <- c('ba' = 'darkgreen', 'da' = 'purple', 'ga' = 'orange', 'other' = 'grey40')
light_colors <- c('#A6C2A1', '#cdacd2', '#ffddac','grey90') %>% set_names(names(dark_colors))

modality_dark_colors <- c('audiovisual' = "#34b44a", 'auditory-only' = "#3b56a6", 'visual-only' = "#e25f26")
modality_light_colors <- c("#7ac585","#7b8ec3","#ec946e") %>% set_names(names(modality_dark_colors))


ebars.x <- function (x, y, sem, length = 0.05, code=0, ...)  {
    arrows(x - sem, y, x + sem, y, angle = 90, code = code, length = length, 
           ...)
}

clean_axis <- function (side, at, tcl = -0.3, labels = at, las = 1, cex.axis = 1.4, 
                        cex.lab = 1.4, mgpy = c(3, 0.6, 0), mgpx = c(3, 0.75, 0), ...) 
{
    if (length(side) > 1) {
        return(invisible(sapply(side, clean_axis, at = at, tcl = tcl, 
                                labels = labels, cex.axis = cex.axis, las = las, 
                                cex.lab = cex.lab, ...)))
    }
    mgp <- mgpy
    if (side%%2) 
        mgp <- mgpx
    invisible(as.matrix(axis(side, at = at, labels = labels, 
                             tcl = tcl, mgp = mgp, cex.axis = cex.axis, las = las, 
                             cex.lab = cex.lab, ...)))
}

midpoint <- function (x) {
    sapply(seq_along(x)[-1], function(ii) {
        (x[ii] - x[ii - 1])/2 + x[ii - 1]
    })
}

round_mean <- function(x, d=1) {
    round(100*mean(x),d)
}

`%within%` <- function (a, b) 
{
    (a >= min(b)) & (a <= max(b))
}

plot_clean <- function (xlim, ylim, x = 1, y = 1, type = "n", xlab = "", ylab = "", 
    cex.main = 1.4, cex.axis = 1.4, cex.lab = 1.4, 
    ...) 
{
    plot(x, y, type = type, axes = F, ylab = ylab, xlab = xlab, 
        xlim = range(xlim), ylim = range(ylim), cex.main = cex.main, 
        cex.axis = cex.axis, cex.lab = cex.lab, ...)
}
```

# 2 Load up AVHuBERT and Human data

```
# the as.numeric is needed to deal with NA values in the spreadsheet that are treated as char
orig_scored <- read_excel("full_data_MaEtAl2025.xlsx", sheet="AVHoriginal_scored_by_phonemes")

var_scored <- read_excel("full_data_MaEtAl2025.xlsx", sheet="AVHvariants_scored_by_phonemes")

hum_scored <- read_excel("full_data_MaEtAl2025.xlsx", sheet="Human_scored_by_phonemes")
```

# 3 McGurk Stimuli

Compare AVH variants with human responses

```
resp_categories <- c("B", "F", "G", "L", "no_cons", "fusion", "all_other")

mcg_by_variant <- aggregate(as.formula(
    sprintf("cbind(%s) ~ StimType + Modality + SubjectID + Aud + Vis",
            paste0(resp_categories, collapse = ',')
    )
), mean, data=var_scored, subset=StimType == 'incong' & Modality == 'audiovisual' & Aud == 'ba' & Vis == 'ga')

mcg_var_agg <- 
    aggregate(as.formula(
        sprintf("cbind(%s) ~ StimType + Modality", paste0(resp_categories, collapse = ','))
    ), round_mean, data=mcg_by_variant)

kable(caption='AVH Variants responses to McGurk',
      fix_agg_columns(mcg_var_agg)
)
```

AVH Variants responses to McGurk


| StimType | Modality | B | F | G | L | no\_cons | fusion | all\_other |
| --- | --- | --- | --- | --- | --- | --- | --- | --- |
| incong | audiovisual | 40.7 | 5.5 | 0.3 | 0.8 | 29.9 | 21.4 | 1.4 |

```
mcg_by_human <- aggregate(as.formula(
    sprintf("cbind(%s) ~ StimType + Modality + SubjectID",
            paste0(resp_categories, collapse = ',')
    )
), mean, data=hum_scored, subset=Modality == 'audiovisual' & Aud == 'ba' & Vis == 'ga')

mcg_hum_agg <- aggregate(as.formula(
    sprintf("cbind(%s) ~ StimType + Modality", paste0(resp_categories, collapse = ','))
), round_mean, data=mcg_by_human) 

kable(caption='Human responses to McGurk',
      fix_agg_columns(mcg_hum_agg)
)
```

Human responses to McGurk


| StimType | Modality | B | F | G | L | no\_cons | fusion | all\_other |
| --- | --- | --- | --- | --- | --- | --- | --- | --- |
| mcgurk | audiovisual | 44.1 | 2.1 | 6.6 | 1.3 | 3.2 | 41.5 | 1.1 |

```
# compare overall non-ba responding
t.test(
    1-mcg_by_human$B, 1-mcg_by_variant$B
)
```

```
## 
##  Welch Two Sample t-test
## 
## data:  1 - mcg_by_human$B and 1 - mcg_by_variant$B
## t = -0.96681, df = 112.53, p-value = 0.3357
## alternative hypothesis: true difference in means is not equal to 0
## 95 percent confidence interval:
##  -0.10398088  0.03578052
## sample estimates:
## mean of x mean of y 
## 0.5592500 0.5933502
```

## 3.1 Plot veridical vs. non-veridical responses

```
barplot(cbind(c(mcg_var_agg$B, 100-mcg_var_agg$B), c(mcg_hum_agg$B, 100-mcg_hum_agg$B)),
        col=c('lightgray', light_colors[1]), border='white', axes=F, names.arg = c('AVH', 'Hum'))
clean_axis(2, 0:2*50)
```

```
mva5 <- unlist(mcg_var_agg[c('B', 'no_cons', 'fusion', 'F')])
mva5 <- c(mva5, 'other'=100-sum(mva5))

barplot(as.matrix(mva5),
        col=rep(c('lightgray', light_colors[1]), c(1, 4)), border='white',
        axes=F)
clean_axis(2, 0:2*50)
mtext(4, line = 0, at = c(25,midpoint(cumsum(mva5))), text=names(mva5), las=1, cex=0.5)
```

# 4 Congruent stimuli

## 4.1 Auditory only ba

```
var_resp_aba <- aggregate(as.formula(
    sprintf("cbind(%s) ~ StimType + Modality + SubjectID + Vis + Aud",
            paste0(resp_categories, collapse = ',')
    )
), mean, data=var_scored,
subset=Modality == 'auditory-only' & Aud == 'ba' & Vis == 'None' & StimType == 'incong')

agg_ao_ba <- aggregate(as.formula(
    sprintf("cbind(%s) ~ StimType + Modality",
            paste0(resp_categories, collapse = ',')
    )), mean, 
    data=var_resp_aba
)

aoba_va3 <- unlist(agg_ao_ba[c('B', 'no_cons')])
aoba_va3 <- 100*c(aoba_va3, 'other'=1-sum(aoba_va3))

barplot(as.matrix(aoba_va3),
        col=rep(c('lightgray', light_colors[1]), c(1, 2)), border='white',
        axes=F)
clean_axis(2, 0:2*50)
mtext(4, line = 0, at = c(aoba_va3[1]/2,midpoint(cumsum(aoba_va3))), text=names(aoba_va3), las=1, cex=0.5)
```

# 5 Change from Ao to AV

```
identical(var_resp_aba$SubjectID, mcg_by_variant$SubjectID)
```

```
## [1] TRUE
```

```
av_ao <- apply(mcg_by_variant[,resp_categories] - var_resp_aba[,resp_categories], 2, m_se)

kable(caption='AbaVga minus Aba',
      fix_agg_columns(av_ao*100) %>% t
)
```

AbaVga minus Aba

|  | mean | se |
| --- | --- | --- |
| B | -44.5542895 | 0.8765821 |
| F | 4.4213514 | 0.3709990 |
| G | 0.2803235 | 0.0726931 |
| L | 0.7743829 | 0.1708046 |
| no\_cons | 20.8356763 | 0.6241430 |
| fusion | 21.2796579 | 0.7594587 |
| all\_other | -3.0371025 | 0.1374777 |

```
dc <- rep(c(dark_colors[1], 'darkgray'), c(3, 1))
h <- unlist(av_ao[1,c('F', 'no_cons', 'fusion', 'B')])*100
hse <- unlist(av_ao[2,c('F', 'no_cons', 'fusion', 'B')])*100
yp <- barplot(horiz=TRUE, height = h, xlim=c(-50,30),
              col=rep(c(light_colors[1], 'lightgray'), c(3, 1)),names.arg=NA,
              border=dc, axes=F
)
clean_axis(1, c(-50,-30,-10,0, 10, 30))
abline(v=0)
ebars.x(h,yp,hse, col=dc)

text(h + sign(h)*1, yp, col=dc, pos=ifelse(h>0,4,2), names(h))
```

# 6 Comparison with Humans

## 6.1 McGurk stimuli

### 6.1.1 Non-veridical responses

```
density_jitter_with_shade <- function(yy, col, cen.x) {
    xp <- density_jitter(yy)
    
    points(cen.x+xp$points, yy, pch=16, col=adjustcolor(col, 2/3))
    
    yd <- xp$density$x
    xl <- (cen.x - 0.05) - (xp$density$y / max(xp$density$y))*.2
    xr <- (cen.x + 0.05) + (xp$density$y / max(xp$density$y))*.2  
    
    lines(x=xl, y=yd, col=col)
    lines(x=xr, y=yd, col=col)
    
    ind <- yd %within% plus_minus(mean(yy), sd(yy))
    polygon(x = c(xr[ind], rev(xl[ind])), y=c(yd[ind], rev(yd[ind])), border=NA, col=adjustcolor(col, 1/3))
}


xph <- density_jitter(1-mcg_by_human$B)
xpv <- density_jitter(x = 1-mcg_by_variant$B)

plot_clean(c(0.75,2.25), 0:1)
density_jitter_with_shade(1-mcg_by_variant$B, 'darkorange', 1)
density_jitter_with_shade(1-mcg_by_human$B, 'darkblue', 2)
clean_axis(2, 0:2/2)
```

```
# range for humans and AVH

range(1-mcg_by_variant$B)
```

```
## [1] 0.3779125 0.8469218
```

```
range(1-mcg_by_human$B)
```

```
## [1] 0 1
```

### 6.1.2 Number of unique phonemes

```
# avh variant raw count
var_raw <- read_excel("full_data_MaEtAl2025.xlsx", sheet="AVHvariants_raw_phonemes")
var_first_ph <- subset(var_raw, Stimtype == 'incong' & Modality == 'audiovisual' & Aud == 'ba' & Vis == 'ga')$First_ph
avhu <- length(unique(var_first_ph))

# human raw count
hum_raw <- read_excel("full_data_MaEtAl2025.xlsx", sheet="Human_raw")

hum_first_ph <- subset(hum_raw, Stimtype == 'mcgurk' & Aud == 'ba' & Vis == 'ga')$First_ph
humu <- length(unique(hum_first_ph))

# dice coefficient for total responses
dice <- length(
    intersect(hum_first_ph, var_first_ph)
) /
length(
    union(hum_first_ph, var_first_ph)
)


barplot(ylim=c(0,20), las=1,
    c(avhu, humu), col=adjustcolor(c('darkorange', 'darkblue'),2/3), border=c('darkorange', 'darkblue'),
    ylab='# Unique Phonemes', names.arg=c("AV HuBERT Variants", 'Humans')
)
```

```
kable(caption='# Unique phonemes and overlap score',
    cbind(avhu, humu, dice)
)
```

# Unique phonemes and overlap score

| avhu | humu | dice |
| --- | --- | --- |
| 18 | 17 | 0.4583333 |

### 6.1.3 Compare frequency of non-veridical response types

```
t.test(
    mcg_by_human$fusion, mcg_by_variant$fusion
)
```

```
## 
##  Welch Two Sample t-test
## 
## data:  mcg_by_human$fusion and mcg_by_variant$fusion
## t = 5.8142, df = 109.1, p-value = 6.149e-08
## alternative hypothesis: true difference in means is not equal to 0
## 95 percent confidence interval:
##  0.1319871 0.2685091
## sample estimates:
## mean of x mean of y 
## 0.4146250 0.2143769
```

```
t.test(
    mcg_by_human$G, mcg_by_variant$G
)
```

```
## 
##  Welch Two Sample t-test
## 
## data:  mcg_by_human$G and mcg_by_variant$G
## t = 4.9502, df = 99.639, p-value = 3.024e-06
## alternative hypothesis: true difference in means is not equal to 0
## 95 percent confidence interval:
##  0.03801709 0.08887644
## sample estimates:
##   mean of x   mean of y 
## 0.066250000 0.002803235
```

```
t.test(
    mcg_by_variant$F, mcg_by_human$F
)
```

```
## 
##  Welch Two Sample t-test
## 
## data:  mcg_by_variant$F and mcg_by_human$F
## t = 3.9606, df = 144.43, p-value = 0.000117
## alternative hypothesis: true difference in means is not equal to 0
## 95 percent confidence interval:
##  0.01705485 0.05103549
## sample estimates:
##  mean of x  mean of y 
## 0.05542017 0.02137500
```

```
t.test(
    mcg_by_human$L, mcg_by_variant$L
)
```

```
## 
##  Welch Two Sample t-test
## 
## data:  mcg_by_human$L and mcg_by_variant$L
## t = 1.1307, df = 126.24, p-value = 0.2603
## alternative hypothesis: true difference in means is not equal to 0
## 95 percent confidence interval:
##  -0.004130549  0.015142890
## sample estimates:
##   mean of x   mean of y 
## 0.013250000 0.007743829
```

## 6.2 Congruent performance

```
var_av_cong <- subset(var_scored, StimType=='cong' & Modality == 'audiovisual')

# var_av_cong %>% split((.)$SubjectID)

var_av_cong$Correct <- NA
for(ii in 1:nrow(var_av_cong)) {
    corr_col <- c(
        'ba' = 'B', 'da' = 'fusion', 'ga' = 'G'
    )[var_av_cong$Aud[ii]]
    
    var_av_cong$Correct[ii] = as.numeric(var_av_cong[ii,corr_col])
}

# AVHubert % Correct AV congruent
kable(caption = 'AVHubert % Correct AV congruent',
      aggregate(
          Correct ~ SubjectID, mean, data=var_av_cong
      ) %$% mean(Correct), col.names = '% Correct'
)
```

AVHubert % Correct AV congruent

| % Correct |
| --- |
| 0.9413887 |

```
# get AV congruent for humans
hum_av_cong <- subset(hum_scored, StimType=='congruent' & Modality == 'audiovisual')

# var_av_cong %>% split((.)$SubjectID)

hum_av_cong$Correct <- NA
for(ii in 1:nrow(hum_av_cong)) {
    corr_col <- c(
        'ba' = 'B', 'da' = 'fusion', 'ga' = 'G'
    )[hum_av_cong$Aud[ii]]
    
    hum_av_cong$Correct[ii] = as.numeric(hum_av_cong[ii,corr_col])
}

# AVHubert % Correct AV congruent
kable(caption = 'Human % Correct AV congruent',
      aggregate(
          Correct ~ SubjectID, mean, data=hum_av_cong
      ) %$% mean(Correct), col.names = '% Correct'
)
```

Human % Correct AV congruent

| % Correct |
| --- |
| 0.972 |

## 6.3 Incongruent non-McGurk

```
var_av_agvb <- subset(var_scored, Aud =='ga' & Vis == 'ba' & Modality == 'audiovisual')

kable(caption='AVH variant GA response to Aga + Vba',
      aggregate(G ~ SubjectID + Aud + Vis, data=var_av_agvb, mean) %>% do_aggregate(
    G ~ Aud + Vis, mean
      )
)
```

AVH variant GA response to Aga + Vba

| Aud | Vis | G |
| --- | --- | --- |
| ga | ba | 0.9092286 |

```
# The human value came from Magnotti & Beauchamp, PLoS CB
```
